# Analysis of Wilms Tumors Using SNP Mapping Array-Based Comparative Genomic Hybridization

**DOI:** 10.1371/journal.pone.0018941

**Published:** 2011-04-22

**Authors:** Lesleyann Hawthorn, John K. Cowell

**Affiliations:** School of Medicine, MCG Cancer Center, Medical College of Georgia, Augusta, Georgia, United States of America; Ohio State University Medical Center, United States of America

## Abstract

Wilms tumor (WT) has been a model to study kidney embryogenesis and tumorigenesis and, although associated with hereditary, cancer predisposition syndromes, the majority of tumors occur sporadically. To analyze genetic changes in WT we have defined copy number changes and loss of heterozygosity in 56 Wilms tumors using high resolution oligonucleotide arrays at a average resolution of ∼12 Kb. Consistent deletions were seen on chromosomes 1p, 4q, 7p, 9q, 11p, 11q, 14q, 16q, and 21q. High frequency gains were seen for 1q and lower frequency gains were seen on 7q and chromosomes 8, 12 and 18. The high resolution provided by the SNP mapping arrays has defined minimal regions of deletion for many of these LOH events. Analysis of CNAs by tumor stage show relatively stable karyotypes in stage 1 tumors and more complex aCGH profiles in tumors from stages 3–5.

## Introduction

Wilms tumor (WT) is the most common kidney cancer in children with an incidence of ∼1∶10,000/year [1]. The tumor contains blastemic, epithelial and stromal components in its tradition triphasic histology. Some tumors also show evidence of further differentiation, providing evidence that WT arises from embryonic precursor cells that have retained a multilineage potential [2]. Although WT is largely a sporadic tumor, rare cases of familial tumors have been described [3]–[5] and WT is also associated with various congential syndromes [6] that predispose to WT, such as the Wilms/Aniridia/GU abnormalities/mental Retardation (WAGR) syndrome [7], Denys-Drash syndrome [8]–[9], Perlman's syndrome [10] and Beckwith-Weidemann syndrome [11]–[12]. In addition, ∼10% of WT patients present with early-onset, bilateral tumors [13], which are considered a hallmark of hereditary tumors fulfilling the Knudson two-hit hypothesis [14]–[15].

The WAGR syndrome was shown to be associated with a deletion involving 11p13 [7], which led to the eventual cloning of the *WT1* gene [16], a zinc finger gene that has been shown to be fundamentally important in kidney development [17] and ∼50% of WAGR patients will develop WT [18]. Loss of heterozygosity (LOH) is considered a manifestation of the Knudson two-hit hypothesis, which explains the exposure of recessive mutations in tumor cells through a variety of mechanisms [19]. In sporadic WT, LOH was also identified at 11p13 [20]–[21], which supported the role of WT1 in these tumors as well. In many cases, however, LOH at 11p13 was accompanied by LOH at 11p15 in sporadic tumors. Interestingly, duplication of the 11p15 region have been identified in BWS patients [22]–[23]. These patients show a 7–10% risk of developing intra-abdominal tumors [24] including WT and some of these patients show loss of imprinting of the maternal 11p15 region which was associated with overexpression of the *IGF2* gene [25]. DDS, which shows high incidence of WT development, was shown to be associated with constitutional WT1 mutations [26].

Although these rare hereditary/syndromic patients implicate 11p13 in Wilms tumorigenesis, only ∼10% of sporadic WT show mutations in *WT1* [27]. This observation prompted surveys of other genetic abnormalities that were present in sporadic tumors. Early chromosome-based analyses identified common abnormalities involving gain of chromosome 1q and loss of 16q [28]–[30]. More extensive genome analysis of WT using individual polymorphic markers [31] defined consistent regions of LOH in addition to those seen on 11p, involving chromosomes such as 7p and 16q. With the advent of array-based comparative genomic hybridization (aCGH), it is now possible to perform whole genome analyses to identify losses, gains and amplifications across the genome at high resolution [32]–[33]. To date, studies of WT have been undertaken using arrays of BAC clones with resolutions of ∼1 Mbp [34] and the Affymetrix 10K SNP mapping array [35]. While these studies have been informative in identifying copy number abnormalities (CNAs), the resolution is still relatively low compared with oligonucleotide arrays currently available. In addition, BAC arrays and most CGH oligonucleotide arrays cannot define LOH that occurs on a background of copy number neutral (CNN) karyotypes [36]. The availability of CGH arrays that carry oligonucleotides defining single nucleotide polymorphisms, however, not only allows detection of CNAs but also LOH, even in the absence of a normal control DNA from the same individual [36]–[37]. By combining these two analyses, a more comprehensive view of the genetic events that have occurred in specific tumors can be defined, since LOH on a CNN background, by exposing recessive mutations, is considered to be equivalent to loss of chromosome regions [36]. We have now performed high-resolution analysis in 56 WT samples using arrays that provide an average ∼12 Kb resolution to generate a comprehensive survey of the CGH/LOH events in these tumors. As a result of this analysis, we have been able to accurately define relatively small regions within individual chromosome arms that show consistent loss of heterozygosity.

## Results

**Figure 1:**
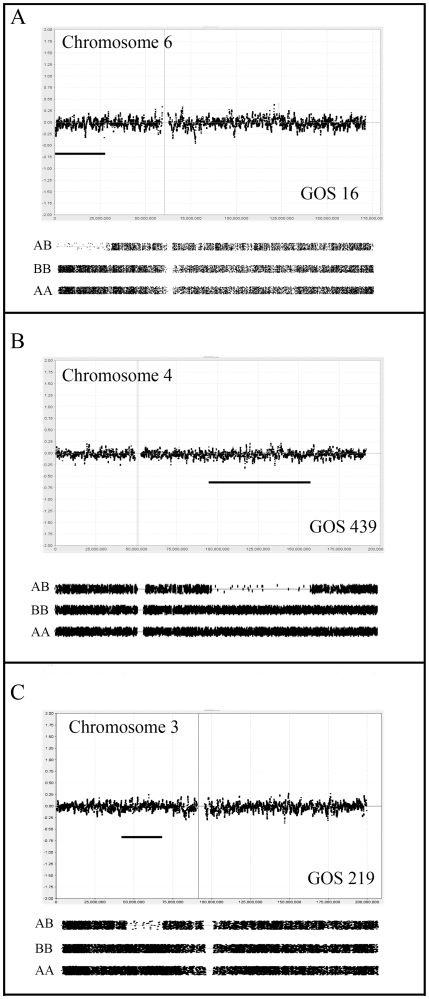
aCGH analysis of copy number neutral LOH events in WT. Upper panels in each case show copy number status for each of the identified tumors (GOS 16, 439, 219) on a Log_2_ scale (0 = no change). The lower panel for each chromosome shows LOH status (see Materials and Methods) coinciding with significant reduction in AB genotypes compared to homozygous AA and BB genotypes. These LOH regions are highlighted by the horizontal bar in the copy number profile. In A-C all LOH events are accompanied by CNN aCGH profiles.

In this study, 56 Wilms tumors were analyzed for copy number abnormalities and loss of heterozygosity using the 250 K Sty1 Affymetrix mapping arrays providing an average resolution of ∼12 Kb. We have previously demonstrated [38] that higher resolution arrays did not provide any significant advantage in CNA detection compared with the 250 K arrays which provide the most cost effective means of analysis [36], [38]. Eight of the tumors did not show any copy number or LOH abnormalities (Supplemental Table S1). Although surprising, this is consistent with previous studies using chromosome-based analyses which similarly defined normal karyotypes in WT [30]. An additional five tumors showed no evidence of CNAs but did show regions of LOH on a CNN background (figure 1). This observation highlights the advantages of using an array platform that can simultaneously identify both CNAs and LOH. The remaining 43 tumors showed structural chromosome abnormalities and/or whole chromosome losses (WCL) and gains (Supplemental Table S1). Tumor DNA was isolated from snap frozen tissue and so the aCGH profiles reflect the total heterogeneity in the original sample.

In this report we first analysed CNAs according to tumor stage, since it has been suggested that higher stage tumors show more extensive genetic instability [39]. The clinical staging system used at the time of diagnosis of the tumors in this study were; Stage I- tumor limited to the kidney and completely excised; Stage II – tumor extends beyond the kidney but is completely excised; Stage III – residual nonhematogenous tumor confined to the abdomen; Stage IV – Hematogenous metastasis; Stage V – bilateral renal involvement.

### aCGH analysis of Stage I/II tumors

Observations within this series of Wilms tumors are consistent with the suggestion that low grade tumors are genetically more stable than higher-grade tumors and reveal fewer CNAs [39]. Of the 12 stage 1 tumors, 5 showed no CNAs and three others showed <3 abnormalities (Supplemental Table S1). GOS 249 showed only CNN LOH for 19q, GOS 207 showed CNN LOH for 6p and loss of whole chromosome 19 and GOS 130 showed CNA_loss_ for the distal part of 4p. Thus, minimal CNA/LOH abnormalities were seen in 8/12 cases (75%). The other 4 tumors showed more extensive CNAs, mostly involving whole chromosome changes (e.g. GOS 101 and GOS 548). In this overall group, since there were relatively few CNAs, no consistent changes were observed, although 1q gain was seen in 2/12 and loss of 1p was seen in only one other cases without 1q gain. Interestingly, in GOS 548, LOH was seen along the length of chromosome 1, but the CNA_loss_ was only identified for distal 1q. The deletion event, therefore, most likely occurred after the duplication of the retained chromosome. Only one tumor (GOS 219) showed CNA_loss_ involving chromosome 11, which, in this case, did not involve the WT1 locus. In this tumor, as described for chromosome 1 above, however, LOH was seen along the length of chromosome 11 but CNA_loss_ only involved distal 11q. One tumor showed whole chromosome 11 loss (GOS 55), accompanied by LOH. 4/12 of these Stage 1 tumors showed CNN LOH. Only one tumor (GOS 548) showed a relatively unstable karyotype with 1p loss and multiple whole chromosome losses. Overall this series of tumors showed relatively stable karyotypes.

**Figure 2 pone-0018941-g002:**
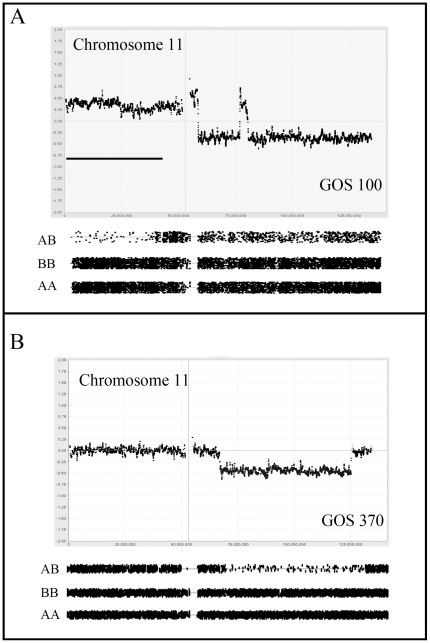
Copy number profiles for abnormalities on chromosome 11. In (A) chromosome 11 from tumor GOS 100 shows a gain of the short arm, which is accompanied by LOH for the distal half of the chromosome arm. LOH is also seen (relative to the AA and BB genotypes) for the long arm which shows a small interstitial region of amplification. In (B) interstitial loss on 11q coincides with LOH.

There was only one example (GOS 100) of a stage II tumor in this series which showed relative instability compared with the majority of stage I tumors. Within this karyotypes, a gain of 1q was observed together with LOH for 6q. A complex series of events involving chromosome 11 resulted in CNA_loss_ and LOH for much of the long arm (figure 2). CNN LOH was present for 11p, including the WT1 locus, in addition to the CNA_loss_ for two distinct regions of 11q. Interestingly, the LOH on 11p was associated with a CNA_gain_. This tumor showed the 16q loss which has been associated with poor outcome (see below), although not accompanied by LOH.

### aCGH analysis of Stage III tumors

Stage III tumors are defined as extending out of the kidney into the abdomen but without hematogenous involvement. Clinically this represents a more aggressive phenotype which, in this series of 16 tumors, is reflected in generally more unstable karyotypes. Despite this trend, four tumors showed minimal changes; GOS 92 showed a normal karyotype, GOS 48 showed loss of a whole copy of chromosome 7, and GOS 16 and GOS 54 showed CNN LOH for 6p (figure 1) and 16p, respectively as the only detectable abnormalities. Loss of 16q was seen in five stage III tumors, in one case as CNN LOH (GOS 54), and another (GOS 404) as WCL with LOH. Two tumors (GOS 52, GOS 97) showed an almost identical loss of Chr16;31624102-qter but without LOH, while another tumor (GOS 11) apparently involved loss of a more distal region of 16q. In fact, both of these breakpoints are located within the centromeric regions of this chromosome leading to loss of the long arm. CNA_loss_ involving 11p was also seen more frequently (5/16) in stage III tumors than in stage I/II tumors. Of the five tumors which showed CNN LOH, three involved the whole chromosome; one (GOS 97) showed CNN LOH and, of the other two showing CNA_loss_, one (GOS 206) showed LOH while the other did not. One tumor, GOS 206, showed exclusive loss of only the distal Chr11:pter-17449758 region, which did not include the WT1 locus. In GOS 11, LOH did not extend to the pter region, but still involved the IGF2 and H19 genes. Two of these stage III tumors (GOS 128, GOS 370) showed CNA_loss_ involving 11q (figure 2), where the minimum region of overlap spanned Chr11;102077451–125002355, which has also been described as a rare event in previous studies as well [40]. Gain of 1q was also notably more frequent (7/14) in stage III tumors, only two of which (GOS 11, GOS 408) were accompanied by loss of 1p.

Of the other notable chromosome events (occurring >2 times) in this series of stage III tumors were losses involving chromosomes 4 and 22 in three cases and chromosome 21 in five cases (Supplemental Table S1). The minimal region of loss on Chr:21 was defined by the CNN LOH event in GOS 11. Also notable was the micro deletion on chromosome 19 in GOS 21 (see below).

### aCGH analysis of Stage IV tumors

Of the 10 stage IV tumors in this series, the frequency of CNAs was generally similar to that seen in the Stage III tumors. One of the tumors in this group, (GOS 231), showed a normal karyotype. Loss of chromosome 11 was seen in 5/10 cases, of which all were associated with LOH. Three of the five involved CNN LOH. These tumors (e.g. GOS 91, 178, 526) tended to have more stable karyotypes compared with the others. In GOS 526, 11p loss involved only 11p13 and not the 11p15 region. Gain of 1q was seen in 4/10 cases and one of these cases was associated with 1p loss. Loss of 7p was seen in 3/10 cases, one including the whole chromosome.

### aCGH analysis of Stage V tumors

Six tumors in this series (∼10%) were from patients with bilateral disease, although only one of the two tumors from each patient was available for analysis. These stage V tumors showed a similar level of instability as stage IV tumors. Of the six tumors in this group, 4 (75%) showed LOH for 11p13, and three were associated with CNN LOH (GOS 541, GOS 566, GOS 586). In three of these cases (GOS 41, GOS 540 and GOS 586), the 11p LOH event was the only abnormality in the aCGH karyotypes. 1q loss was observed in only 1/6 tumors (GOS 12), with accompanying CNA_loss_ and LOH of 1p.

### Overall analysis of CNAs in Wilms tumors

**Figure 3 pone-0018941-g003:**
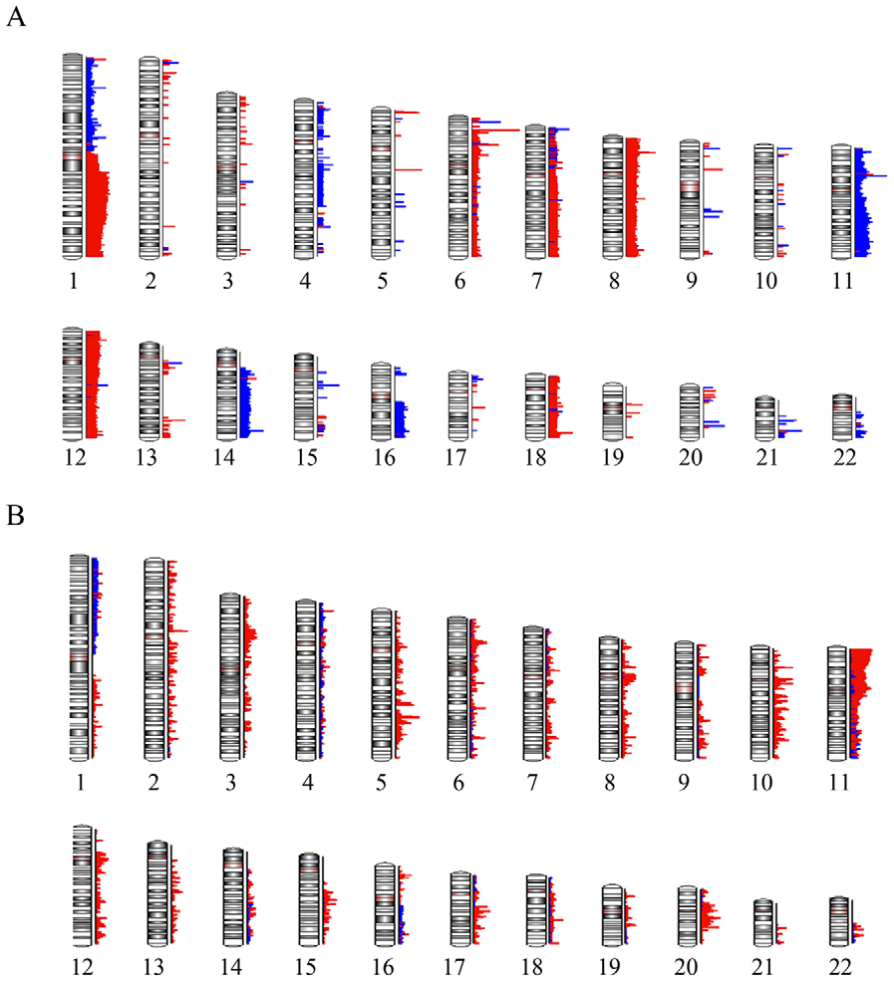
Summary of copy number losses and LOH in the Wilms tumor series. Distribution of copy number gains (red) and losses (blue) throughout the genome of the Wilms tumor series (A). Distribution of LOH events throughout the WT genomes (B). Copy number neutral LOH events are shown in red. LOH associated with copy number loss is shown in blue.

To define consistent CNAs in WT, independent of tumor stage, we combined the data from all 56 tumors to establish relative frequencies of specific CNA/LOH events, and also to define minimal regions of loss and gain associated with these CNAs. Segmental chromosome losses were infrequent on many (Supplemental Table S1) and only chromosomes 1, 4, 11, 14, 16 and 17 showed abnormalities in >4 tumors. In addition, whole chromosome losses were observed >3 times only for chromosomes 4, 6, 11, 14 and 21. A summary of the CNA/LOH events on a genome-wide, low-resolution plot is shown in Figure 3. Loss of heterozygosity, coincident with CNA_loss_ was seen on average in only ∼60% (37/61) of cases. As we have described previously [38], this observation likely reflects either heterogeneity in the tumor or higher ploidy levels in the cells where loss of chromosomal material on a diploid background results in LOH, whereas a single chromosome loss on a tetraploid background does not. The frequency with which whole chromosome losses also resulted in LOH, however, was much lower, possibly reflecting the chromosome instability seen in higher ploidy cells.

**Figure 4 pone-0018941-g004:**
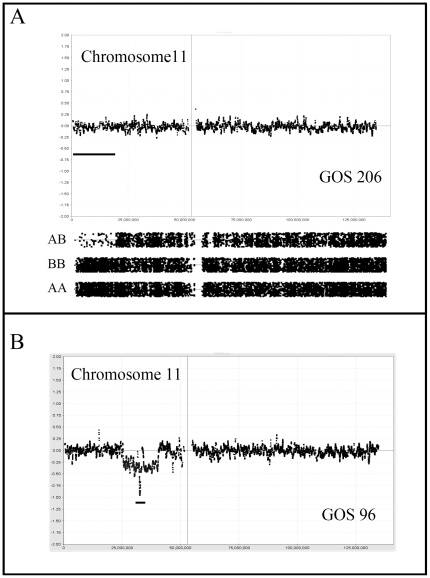
Genetic changes involving the WT1 and WT2 loci. In (A) CNN LOH is identified on chromosome 11 in GOS 206 which is restricted to the distal 11p15 region containing WT2. In contrast, in GOS 96 (B) the deletion is confined to the WT1 locus where one chromosome carries an extensive deletion and the other homolog carries a micro deletion (indicated by the bar) spanning the WT1 locus.

Chromosome 11 showed involvement in CNA_loss_ in 19 tumors of which seven involved the whole chromosome (4 with LOH and 3 without). Of the 12 with subregional CNA_loss_, 10 were accompanied by LOH, but 6 (50%) of these did not involve the 11p15-p13 region. Of the six tumors showing 11p deletions, five did not extend to the 11p15 region containing the *IGF2/H19* genes (the WT2 locus). An additional 18 tumors showed CNN LOH, where 5 showed involvement of the whole chromosome and 5 were confined to 11p regions that did not contain the WT1 locus (figure 4). Of the remaining seven LOH events, six spanned the WT1/WT2 loci and only one showed LOH that excluded the WT2 locus. Of the 12 tumors showing sub regional LOH, none involved the long arm, in contrast with the tumors with CNA_loss_, where 50% involved the long arm. Since WCL was seen in seven cases, involvement of 11q occurred in 15 tumors (∼28%). A compound deletion was seen in GOS 96 resulting in a homozygous deletion of the WT1 locus (figure 4). Of the tumors showing LOH that extended to 11qter, the minimal region of overlap (MRO) was defined as Chr11;102077451–125002355, by tumors GOS 128, (Chr11:102077451-qter) and GOS 370 (Chr11; 66706205–125002355). The other interstitial deletion seen in GOS370, which did not extend to qter, reduced the MRO to Chr11:102077451–125002355. Overall, therefore, 37 tumors (∼66%) showed loss or LOH involving chromosome 11.

Gain of the long arm of chromosome 1 has been associated with poor outcome [40]. In this series, 22 tumors (∼40%) showed a gain of 1q which, with the exception of GOS 26, involved the entire long arm. Loss of a whole copy of chromosome 1 was never seen, and only one tumor (GOS 548), showed LOH of Chr:1 on a CNN background. Chromosome 1 showed loss of the short arm in seven tumors, of which four were accompanied by a gain of the long arm. 5/7 CNA_loss_ events were accompanied by LOH and, in all but GOS 11, involved loss of the entire chromosome arm.

**Figure 5:**
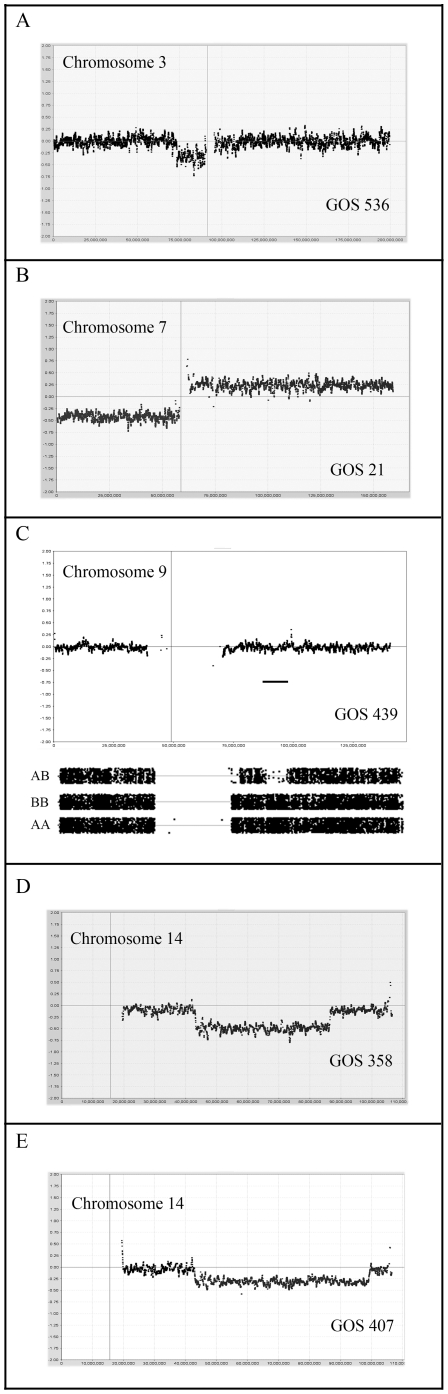
Distinct genetic events in WT. In (A) a small deletion is observed on chromosome 3p in GOS 536 which defines the MRO for losses on this chromosome. In (B) loss of 7p and gain of 7q (iso-7q) in GOS 21 reflects a common event in WT. The CNN LOH event in GOS 439 (C) involves a small region on 9q which defines the MRO for losses in this series of WT. The overlapping deletions on chromosome 14 in GOS 358 (D) and GOS 407 (E) define the MRO of losses on this chromosome.

The short arm of chromosome 3 showed losses in six tumors, of which three represented CNN LOH (figure 5) and two involved WCL. The common region of loss involved Chr3:40029744–53237440.

Chromosome 16 has also been implicated in frequent LOH [31], [41]–[42], which has been related to clinical outcome [43]–[45]. One tumor in this series (GOS 404) showed whole chromosome 16 loss accompanied by LOH and six other tumors showed subregional deletions involving only the long arm, of which only 2 showed coincident LOH. The breakpoints giving rise to the deletions clustered in two distinct regions, one at ∼31 Mbp and the other at ∼45 Mbp, both extending to 16qter. Interestingly, only the deletions showing the 45 Mbp-qter loss showed LOH, although both breakpoints occurred within the large heterochromatic regions on either side of the centromere of this chromosome. Two other tumors (GOS 54 and GOS 439) showed CNN LOH for non-overlapping regions of Chr16. Gains involving any part of Chr16 were not observed.

Losses on chromosome 7p (figure 5) have also been described in WT [46] and were observed in six tumors in this cohort. Of the 4 tumors showing subregional deletions, three were accompanied by LOH, although neither of the two cases of WCL showed LOH. The minimal LOH region on 7p was defined by GOS 120 as Chr7:pter-46805718. In one case (GOS 44), as we reported previously [47] using focused analysis of polymorphic loci, a homozygous deletion was seen spanning the Chr7:14474403–18712248 interval. Loss of 7p was only seen in the stage III/IV tumors. Gains of whole chromosome 7 were seen in four tumors but no examples of CNN LOH for Chr7 were observed. From the three tumors showing subregional gains, the minimal region involved Chr7:69218189-qter.

Loss of 9q has been reported to be related to increased tumorigenicity [35]. In the current series, five tumors showed losses involving chromosome 9. In three of these cases, the whole chromosome was lost, of which one was associated with LOH and one represented CNN LOH. In three other tumors, subregional losses on the long arm were observed, two as CNN LOH, where the minimal region of loss was defined by tumor GOS 439, as Chr9;84192301–93960316 (figure 5), which overlaps with the Chr9:85684008–112037919 region defined by Ruteshouser et al [48]. This ∼10 Mbp minimal region is a relatively gene dense region carrying genes for protein kinase such as *TRKB* and *SYK*, cell cycle related proteins *GAS1* and *CDK20*, and the *RASEF* member of the RAS family of proteins.

Losses involving chromosome 14 were seen in ten tumors. Six of these involved the whole chromosome, of which 50% showed LOH. Subregional deletions were seen in the other 4 with Chr14:42827908–79037514 as the common region of overlap, defined by tumors GOS 407 and GOS 358 (figure 5).

Three tumors showed CNN LOH for chromosome 6, where the common region of overlap, defined by tumors GOS 16 (figure 1) and GOS 576, was Chr6:24916262–26252770. This region contains a histone gene cluster and a solute carrier 17 (*SLC17*) gene cluster. A non-overlapping deletion on 6q, which involved LOH, was seen in GOS 100.

In a recent report by Yuan et al [35], a novel region of LOH on chromosome 4q was defined in 8% of tumors. In our series, ten tumors showed losses involving chromosome 4 (figure 1). Half of these deletions involved the whole chromosome, of which three were accompanied by LOH. Of the 5 tumors showing sub regional 4q losses, four cases involved CNA_loss_ of which two showed LOH and one showed CNN LOH. The minimal region of overlap for these deletions/LOH (excluding the non-overlapping 4p deletion in GOS 404) involved the Chr4:92539047–129243723 interval which includes the 2.4 Mbp region described by Yuan et al [35]. This region does not encompass the *FBXW7* gene at Chr4:153242411–153456185, which was shown recently to be mutated in a small (<4%) number of WT [49].

Gains involving chromosome 12 were seen in nine tumors, of which five involved the whole chromosome. The minimal region of gain was Chr12:26989469–34451374 which includes a member of the PI3K kinase family.

**Figure 6:**
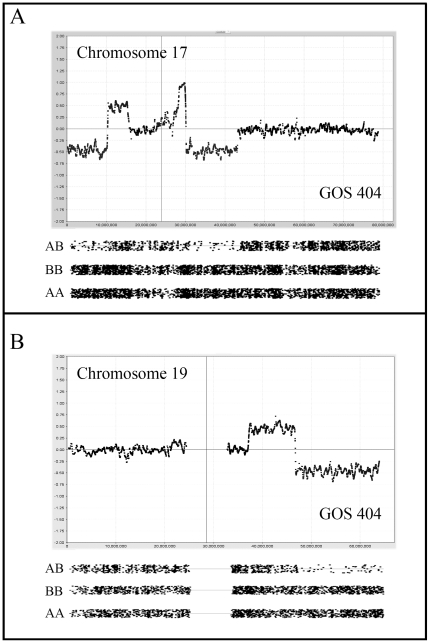
Complex CNAs in WT. In (A) two regions of deletion on chromosome 17 are seen in GOS 404, in both cases accompanied by amplification at adjacent regions. LOH is seen for the two deleted regions. In (B) deletion of the distal part of 19q is seen in GOS 404 which is accompanied by LOH with a preceding region of gain on 19q.

The TP53 gene is located on the short arm of chromosome 17 (Chr17:7577861–7590863). Mutations in this gene occur infrequently and are more often seen in rare tumors with anaplastic histology [50]. In the current series, CNA_loss_ on Chr17 was seen in six tumors, of which one involved whole chromosome loss. The other five involved losses of 17p all of which included the *TP53* gene. The MRO was defined by GOS 404 (figure 6) which spanned Chr17:pter-10157411 and was the only tumor accompanied by LOH. Interesting, in GOS 404, a second region of CNA_loss_, also with LOH, was seen more proximally on the short arm (figure 6). CNN LOH was also seen in two other tumors (GOS 439, GOS 536) but involved the long arm of the chromosome. The MRO for these 17q events was Chr17:66017832–72094821.

Chromosome 19 (figure 6) was infrequently involved in CNA_loss_ although one tumor (GOS 21) showed a micro deletion spanning Chr19:48104372–48373067, which was within a common deleted region. Losses involving chromosome 21 occurred in 9 tumors (5 as WCL), where the maximum common region of overlap in the other four was Chr21:22871547–31525365. Similarly, CNA_loss_ of chromosome 22 was observed in seven tumors of which two involved subregional losses accompanied by LOH, where the smallest region of loss was defined by GOS 206.

Chromosome 22q LOH has also been associated with poor outcome [51]. In the current series we identified LOH involving 22q in 7 tumors of which 5 involved the whole chromosome. Of the other two, the minimal deletion was defined as Chr22:40456765-qter.

### Analysis of aCGH profiles in tumors with different histology

Within this series of tumors, histology subtype was only available from 28/56 of the cases (50%). Overall there were 22 cases with reported favorable histology (FH) and 6 with unfavorable histology (UH). Although these numbers are too small to draw definite conclusions, there appears to be an excess of three chromosome abnormalities that are associated with poor survival in the unfavorable histology group. Thus, 4/6 (66%) of UH cases showed gain of chromosome are 1q compared with 6/22 (27%) in the confirmed FH group. Similarly, 3/6 UH (50%) tumors showed losses of chromosome 16q compared with only 1/22 (4.5%) of FH tumors. Finally, losses of chromosome 11 were also seen in 50% of UH tumors compared with 7/22 (32%) in FH tumors. All of the other molecular changes frequently seen in this series of WT (see above) were not found in UH tumors and appeared to be only associated with FH tumors. Clearly given the small numbers of tumors for which histological sub-typing was available, these observations only indicate trends but molecular markers associated with poor outcome tumors are consistently observed at higher frequency in UH tumors which is also a marker for poor outcome.

## Discussion

In this analysis of Wilms tumors, we have combined data from LOH events on a CNN background with LOH associated with CNA_loss_ to provide a comprehensive analysis of genetic abnormalities at high resolution (∼12 Kb) using SNP mapping arrays. In many cases, comparison of overlapping CNA/LOH has highlighted subregions within chromosomes that are consistently lost, potentially defining the location of tumor suppressor genes. Until recently, LOH studies in WT had traditionally involved analyses of microsatellite markers, although using this approach only low resolution analysis was possible because of the small number of loci studied. As such, integration of this data with higher resolution studies is relatively imprecise. In addition, not all microsatellite loci were informative in LOH analyses due to constitutional homozygosity. In some cases, because of constitutional homozygosity, the LOH status was not retrievable at critical loci, which limited resolution and restricted the number of cases available for correlative analyses. This technical shortcoming is largely overcome by the SNP mapping arrays which do not require the availability of constitutional DNA from the same patient [36] and, because of the high density of polymorphic markers on the array, the extent of LOH can be relatively accurately determined [37]. As a result of our analysis of WT, we have defined regions throughout the genome that show consistent LOH. The two hit hypothesis predicts exposure of recessive mutations in critical tumor suppressor genes as a consequence of this LOH [14], [19]. In this series of WT, LOH events (generated by whatever means) was seen in 45% of tumors.

LOH involving the short arm of chromosome 11 is a frequent event in sporadic WT [21] and reflects the importance of the WT1 (11p13) and WT2 loci (11p15) in tumorigenesis. It has been suggested that the WT1 locus in 11p13 is more frequently affected in syndrome related tumors [35], especially where hereditary deletions (in WAGR) or mutations (in DDS) are seen. Bilateral tumors are also considered to be more likely to be the consequence of genetic predisposition [15], which is supported by the fact that 75% of stage V tumors in this series showed LOH for 11p extending to the WT1 and WT2 loci. In two cases, these LOH events were present as the only abnormalities in the tumor, suggesting an important role for this event in these tumors. Only one other tumor (stage IV) showed LOH for 11p as the only aCGH/LOH abnormality in this series, and only 2/13 stage I/II tumors carried this abnormality.

Mutations in *WT1*, however, are infrequent in sporadic WT [52] supporting an important role for the *H19* and *IGF2* imprinted genes in 11p15, where LOH results in preferential loss of the paternally silenced genes resulting in their over expression [35]. The *IGF2* gene is located in Chr11:2150348–2162341, and H19 is located ∼200 kb distal to that. In our series, of the tumors showing CNA_loss_ involving 11p, only three (50%) showed exclusive loss of the region harboring the *IGF2* and three others showed loss restricted to the WT1 locus. In contrast, of the 12 tumors showing CNN LOH, eight involved both the IGF2 and WT1 loci, and the other four showed exclusive loss of the IGF2 locus. Thus, there appears to be two consequences of 11p loss. In tumors showing CNA_loss_ as a means of generating LOH, and hence only one copy of the IFG2 locus, 50% show loss that does not involve *IGF2* and the other 50% involved both.

Despite the now well known involvement of loci on 11p in Wilms tumorigenesis, deletions on the long arm of chromosome are also common and have been associated with an increased risk of relapse [53]–[54]. In the present series, 14% of the tumors showed sub regional CNA_loss_ involving 11q, but overall in 28% of tumors, if whole chromosome losses are included. These frequencies are consistent with the 19% reported by Wittmann et al [53]. The high resolution capabilities of the 250 K SNP array used in our studies defined Chr1:102077451–125002355 as the MRO. In the study by Wittmann et al [53], although only 11 SNPs along the length of the chromosome arm were used, several tumors showed LOH confined to the interval defined by markers D11S2000 and D11S925. This region spans Chr11:105,659,004–120,728,211, which is consistent with our analysis,.

Genome-wide scans for LOH also identified 16q as a relatively frequent event in WT [31], [41]. Although different studies used different microsatellite markers, the overall conclusion was that the whole arm of the chromosome was usually involved, which related to the frequent observation of unbalanced chromosome translocations involving chromosome 16 in WT [28]–[29], [55]–[56]. Following early evidence that LOH for 16q was related to poor outcome, recent studies using large sample sizes confirmed that LOH on 16q is related to early relapse and poorer survival [43]–[45], although no specific culprit gene within this chromosome arm has been identified. In the present study, LOH for chromosome 16q, in any form, was seen in 12 tumors, although only associated with LOH in 50% of cases. Thus, the overall frequency of CNA_loss_/LOH involving of 16q is ∼16% which is comparable to the LOH frequency of ∼17% in large cohort analyses [40], [43]–[44]. Also, small numbers of markers spaced along the length of the chromosome arm may overlook small regions of LOH. An advantage of microsatellite analysis, however, is that LOH can be identified if, as suggested by Grundy and colleagues [43]–[44], there was “a definitely reduced intensity” of the minor allele. This criterion allows LOH to be detected even if there is heterogeneity, or presence of normal cells, in the tumor sample. In the SNP mapping arrays, heterozygosity contributed by these complications would tend not to report LOH, which might account for the slightly lower frequency of 16q LOH in our series, although after combining CNA_loss_ and CNN LOH, there is no difference in incidence between different studies. The high-resolution analysis provided by the SNP mapping arrays, for this and other chromosomes, offers the possibility of detecting critical sub regions of 16q. In tumors with CNA_loss_, however, loss of 16q involved the entire arm in 5/5 cases with all breakpoints located within the heterochromatic regions flanking the centromere. The same was true of breakpoints involving gain of 1q where, although different breakpoints are apparently identified, all occur within the large heterochromatic regions on either side of the centromere on this chromosome as well. What was striking on chromosome 16, however, was the fact that CNN LOH in one tumor (GOS 439) involved only a sub region of 16q, defining the MRO as Chr16:45086927–53627194, albeit based on a single tumor. In the study by Wittmann et al [40], there was also evidence from a small numbers of tumors that suggested a region involving ∼Chr16:54.4–55.5 Mbp, although only two microsatellite markers were used in this region to assess LOH. Nonetheless, these observations combined, suggest involvement of an ∼10 Mbp region which contains at least 40 annotated genes.

According to the two hit hypothesis [14], bilateral tumors are more likely to be related to hereditary predisposition. In our series there were only 6 confirmed stage V tumors and genetic instability was very similar to that seen in the stage III/IV tumors. One of the tumors in the series, GOS 358, was classified as hereditary since the sister of this patient also developed WT. In this case there were only minimal genetic changes with LOH only for chromosome 11 and regions on chromosome 14. This is consistent with the observations by Ruteshouser et al [48] who demonstrated that LOH frequency in tumors from patients that carried either constitutive mutations in *WT1*, or acquired *WT1* mutations in their tumors, showed a decreased loss of heterozygosity (except for the WT1 locus) using a genome microsatellite analysis compared with tumors that were wild-type for *WT1*. In our series, several tumors (GOS 566, GOS 178, GOS 543, GOS 586, GOS 504) showed LOH involving the WT1/WT2 loci on chromosome 11 as the only abnormality detected by the SNP arrays, although the *WT1* status in these tumors is not known and none were recorded as hereditary. Several other tumors with LOH of 11p showed only minimal, if any, additional CNA/LOH changes. These observations are consistent with the idea that loss of *WT1/2* is a significant driver event in Wilms tumorigenesis that does not require significant additional changes, whereas tumors without 11p abnormalities show more complex molecular karyotypes. The presence of abnormalities involving only 11p was more prevalent in the stage V tumors, which is consistent with this suggestion. In one of these cases the 11p deletion was confined to the WT1 locus.

Several tumors apparently showed normal karyotypes which was independent of stage and histology, since there were representatives of this class in all but the stage V tumors (see above), and at least one case from a tumor with unfavorable histology. It appears, therefore, that molecular profiles are becoming essential in the characterization of these tumors rather than staging. For Wilms tumors, the staging system is based on clinical parameters, and so the timing of diagnosis could provide a lower or higher stage, which may not be related to the molecular events that have occurred within the tumor cells.

In addition to the common LOH events seen in WT involving 1p, 7p, 11p, and 16q, several other regions of the genome have been highlighted in individual studies. Although mutations in specific genes have been described, their incidence is still relatively low. The *CTNNB1* (β-catenin) gene located on chromosome 3 (Chr3:41240942–41281939), for example shows mutations in >10% of WT [56] and more often in tumors that carry *WT1* mutations. In our study, we defined the Chr3:40029744–53237440 interval as the MRO which encompasses *CTNNB1*, suggesting this may be the driver for the rare LOH events on chromosome 3.

In summary, we have undertaken a detailed analysis of a relatively large cohort of WT using high resolution arrays that define CNAs and LOH to define regions of consistent losses and gains throughout the genome. As a result of this analysis, and combined with other studies, a detailed genomic landscape has been defined which provides a platform to identify genes related to the development and progression of WT and which might serve as unequivocal markers for poor outcome.

## Materials and Methods

Tumor samples were collected immediately following surgical resection during the period 1982–1992 and snap frozen in liquid nitrogen. At the time of collection the diagnosis of WT was confirmed, although in some cases the specific stage was not recorded. Unfortunately, it has also not been possible to recover this information retrospectively from some of the anonymized legacy samples. Approval to use the DNA samples and a waiver of consent was obtained from the Roswell Park Cancer Institute IRB where the CGH analysis was performed. Verbal informed consent for the use of these tumor samples was obtained from parents of children involved, which was the accepted procedure at the Hospital for Sick Children, London, UK, during the collection period. DNA was prepared from the whole tumor sample from snap frozen tissue using standard phenol/chloroform extraction procedures.

### Analysis of 250K Genotyping Arrays

For the analysis of individual tumors, allele signal summaries and genotypes were generated from the .CEL files with the command-line program apt-probeset-genotype (v. 1.6.0; the Affymetrix Power Tool [APT] package). The files were processed using the Bayesian Robust Linear Model with Mahalanobis distance (BRLMM) algorithm implemented in APT [57] with a quantile sketch normalization of 50,000 points and no background correction. Array data from the International Hap Map project was used in the normalization and as baseline reference signals for copy number (CN) estimations. Copy number was estimated with the apt-copynumber-pipeline program [58] with Gaussian smoothing at 0.1 Mb. These basic functionalities are also available in the CNAT4 software [59]. Copy number is estimated as a log-sum of the normalized sample allele signals (S) against those of the reference set (R): Log Ratio = log_2_ (S_a_/(R_a_+R_b_)+S_b_/(R_a_+R_b_)) where a and b refer to the alternative SNP alleles. A detailed review of considerations for CNA analysis is described elsewhere [37]. Losses and gains were called where >20 contiguous probes showed variations in log_2_ ratios of +/− 0.3. The Gaussian smoothed log_2_ were then viewed using aCGHViewer [60] which separates each chromosome into an individual panel to explore in greater detail.

LOH was estimated from the sample genotypes by a Hidden Markov Model using the SNP-wise heterozygosity rate of the reference population and a genotyping error of 0.02 (default) to estimate the prior. The transition decay parameter (describing the influence of the LOH state of neighboring SNPs) was set to 10 Mb. The state prediction of "LOH" versus "retention of heterozygosity" (1 or 0) by the HMM was used to map regions of empirical LOH as described in detail elsewhere [37]. From our extensive analysis of LOH parameters that represent true LOH in the absence of a matched normal control, using our custom algorithm, an LOH score of >50 can typically be used reliably to demonstrate LOH. For LOH visualization we used two complementary approaches. Firstly, the LOH score for the loss of heterozygosity is calculated by multiplying the reference homozygous rate for a contiguous stretch of SNPs using an in-house script, that has been classified as having likely LOH using the Hidden Markov Model that is incorporated into CNAT 4.0.1. The reference homozygosity rate is obtained from the allelic frequencies derived from 90 phenotypically normal individuals from the HapMap project (data available at http://www.affymetrix.com). The calculated p-value represents the likelihood that a contiguous stretch of SNPs would be homozygous by chance alone, based on the allelic frequencies defined by the control samples. The LOH Score is then calculated using the -Log_10_(pLOH_sample_). The .CHP files from the BRLMM analysis was also processed in Partek Genomics Suite v6.4 where the A/B allele ratios were used to generate ideograms for LOH as shown in figures 1–5. We used an unpaired analysis where the probability of observing a heterozygous SNP in a region of LOH is the genotype error rate. In a region without LOH, the probability of observing a heterozygous SNP is estimated using the observed frequency from the baseline samples. The heterozygosity rate (HET rate) is calculated as the number of AB calls/total number of calls, where low het rates imply LOH. By default the frequency of heterozygous calls for a normal region is 0.3. We used a het rate of <0.07 for calling LOH events. The allelic ratios for the SNPs at each reported event were graphed and visually examined and any reported regions that were found in areas of poor probe density or close to centromeres were identified. The analysis limits the number of markers on the LOH fragment to a minimum of 20. The LOH data was then assimilated and compared with copy number data.

## Supporting Information

Table S1 **Summary of losses, gains and LOH in 56 WT samples.** Tumors are listed according to stage except where this was not available (NA). Segmental (Seg) and whole chromosome losses or gains (WCL, WCG) which were accompanied by LOH are shown in bold. For some tumors histology was defined as either favorable (FH) or unfavorable (UH) and deceased individuals are defined as (*).
(DOCX)
