# Supplementary material for: Analysis of Wilms Tumors Using SNP Mapping Array-Based Comparative Genomic Hybridization
Source: PLoS One. 2011 Apr 22;6(4):e18941. doi: 10.1371/journal.pone.0018941 (PMC3081321; doi:10.1371/journal.pone.0018941)
Supplement: Table S1 — Summary of losses, gains and LOH in 56 WT samples. Tumors are listed according to stage except where this was not available (NA). Segmental (Seg) and whole chromosome losses or gains (WCL, WCG) which were accompanied by LOH are shown in bold. For some tumors histology was defined as either favorable (FH) or unfavorable (UH) and deceased individuals are defined as (*). (DOCX) [file pone.0018941.s001.docx]

**Supplemental Table S1. Summary of losses, gains and LOH in 56 WT samples.** Tumors are listed according to except where this was not available (NA). Segmental (Seg) and whole chromosome losses or gains (WCL, WCG) which were accompanied by LOH are shown in bold. For some tumors histology was defined as either favorable (FH) or unfavorable (UH) and deceased individuals are defined as (*).

| TUMOR | Hist | Seg. Losses | Seg.Gains | WCL | WCG | LOH |
| --- | --- | --- | --- | --- | --- | --- |
| **Stage I** |  |  |  |  |  |  |
| GOS55 | FH | Chr15:24740085-48726270 |  | **11,** 14, | 3, 6, 7, 8 |  |
| GOS88 |  | ******** | ******** | ******** | ******** | ******** |
| GOS90 | FH | ******** | ******** | ******** | ******** | ******** |
| GOS101 | UH* | Chr20:pter-21025215 | Chr1:144255274-qter | 3, 4, 5, **6,** 14, 21 | 12, 17, 18 | ******** |
| GOS119 |  | ******** | ******** | ******** | ******** | ******** |
| GOS130 |  | ******** | Chr4:pter-9845816 | ******** | ******** | ******** |
| GOS207 | FH | ******** | ******** | 19 |  | Chr6:pter-57701929 |
| GOS219 | FH | **Chr11:78694008-qter** | Chr1:144262872-qter | ******** | 12, 18 | Chr3:40029744-63487802  Chr11 |
| GOS249 | FH | ******** | ******** | ******** | ******** | Chr19:41656621-qter |
| GOS360 | FH | ******** | ******** | ******** | ******** | ******** |
| GOS399  Hered | FH | ******** | ******** | ******** | ******** | ******** |
| GOS548 |  | **Chr1:pter-120967708**  Chr12:91387427-qter  Chr17:pter-20649281 | Chr12:pter-91396989 | 3, 4, 6, 11, 14, 15, 16, 18,21, 22 | 7, 8, 20 | Chr1 |
| **Stage II** |  |  |  |  |  |  |
| GOS100 | FH | **Chr6:67569490-qter**  **Chr11:58323281-76689952**  **Chr11:80308347-qter**  Chr16:31476343-qter | Chr1:144255274-qter  Chr11:pter-58368275  Chr11:76646423-80321749 | ******** | ******** | Chr11:pter-36389195 |
| **STAGE III** |  |  |  |  |  |  |
| GOS11 |  | **Chr1:pter-96591044**  **Chr11:3351536-qter**  **Chr16:45086927-qter** | Chr1:144255274-qter | ******** | ******** | Chr21:28624694-31525365 |
| GOS16 | FH | ******** | ******** | ******** | ******** | Chr6:pter- 26252770 |
| GOS21 |  | **Chr7:pter-57834342**  Chr19:48104372-48373067 | Chr1:143916898-204661633  Chr7:57828265-qter | ******** | ******** | Chr11:pter-45744670 |
| GOS48 |  | ******** | ******** | 7 | ******** | ******** |
| GOS51 | FH | Chr21: 22871547-qter | Chr7:14785697-qter  Chr12:pter-73193477  Chr13:89784460-qter | ******** | 9, 18 | ******** |
| GOS52 | UH | Chr16: 31624102-qter | Chr1:143916898-qter | 17, 21 | 6, 9, 10 | ******** |
| GOS54 | FH | ******** | ******** | ******** | ******** | Chr16:1675672- 5357716 |
| GOS92 |  | ******** | ******** | ******** | ******** | ******** |
| GOS97 | UH | Chr16:31488031-qter | ******** | ******** | 12 | Chr:11 |
| GOS126 | FH | Chr2:23811668-37770867  Chr9:77266560-103624313 | ******** | 22 | ******** | Chr3:pter-53237440 |
| GOS128 | FH | **Chr11:pter-44903980**  **Chr11;102077451-qter** | Chr1:142758415-qter  Chr4:pter-22195630 | 21 | ******** | ******** |
| GOS206 | FH | **Chr22:40456765-qter** | Chr1:143916898-qter | ******** | ******** | Chr11:pter-17449758 |
| GOS370 |  | **Chr11:66706205-125002355** | ******** | ******** | ******** | ******** |
| GOS404 | UH | **Chr4: 8321220-53513854**  **Chr17:pter-10157411**  **Chr17:29864353-43119234**  **Chr19:46596236-qter** | Chr4:pter-8321220  Chr8:pter-105900459  Chr17:10142840-15848909  Chr17:22682442-29866716  Chr19:37005651-46612605 | **16** | 13, 18 | ******** |
| GOS407 | UH* | Chr14:42827908-99117470 | Chr1:141644337-207312482 | 4, 11 21 22 | ******** | ******** |
| GOS408 | UH | **Chr1:pter-116958895** | Chr1:116945913-qter | **4, 9, 11, 14** | ******** | ******** |
| **Stage IV** |  |  |  |  |  |  |
| GOS44 | FH | **Chr7:pter-69218189** | Chr7:69218189-qter  Chr10:88227675-qter | 7 | 8, 12 | Chr18 |
| GOS91 |  | ******** | ******** | ******** | 12, 18 | Chr:11 |
| GOS96 | FH* | **Chr11:25212967-40182957**  Chr21:27209349-28911573 | ******** | ******** | ******** | Chr3:pter-71403588 |
| GOS120 | FH | **Chr7:pter-46805718** | Chr1:144281984-qter  GOS126 | ******** | ******** | ******** |
| GOS142 |  | **Chr2:228212747-234723089**  Chr21:pter-29174433 | Chr1:143887291-186518785 | ******** | 8 | ******** |
| GOS178 | FH | ******** | ******** | ******** | ******** | Chr11:pter-5875537 |
| GOS231 | FH | ******** | ******** | ******** | ******** | ******** |
| GOS358 | FH* | **Chr14:41996736-79037514** | Chr1:141644337-qter | 9 | 3, 6, 8, 18 | Chr11 |
| GOS526 |  | ******** | ******** | ******** | ******** | Chr10:36931034-105733788  Chr11:30048657-61358484  Chr15:46950338-61002704  Chr15:91654150-99815514 |
| GOS550 |  | **Chr1:pter-117007859**  Chr15:68945904-73677667 | Chr1:116979999-qter | 4, 11, 14 | ******** | Chr9 |
| **Stage V** |  |  |  |  |  |  |
| GOS12 |  | **Chr1:pter-143916898**  **Chr22:17012376-qter** | Chr1:143887291-qter | ******** | ******** | ******** |
| GOS41 |  | **Chr2: 230109326-qter**  **Chr12: 70966468-qter**  **Chr20:pter- 24962206** | Chr6:pter-64586994  Chr12: 26989469-70895908  Chr15:7293374-qter | ******** | 3, 7, 8, | Chr11:pter-47485613 |
| GOS66 | FH | ******** | ******** | ******** | ******** | Chr11:pter-51049446 |
| GOS504 |  | **Chr11:25840913-35600517** | ******** | ******** | ******** | ******** |
| GOS536 |  | Chr3:72790543-89566958  Chr4:53815219-129243723  Chr14:25569587-qter  Chr17:pter-41361241 | Chr8:pter-80225538  Chr10:97230090-qter  Chr12:pter-34451374 | 22 | ******** | Chr8:95327241-qter  Chr12:42685286-qter  Chr17:43972018-qter |
| GOS586 |  | ******** | ******** | ******** | ******** | Chr11:pter-47162658 |
| **NA** |  |  |  |  |  |  |
| GOS15 |  | Chr1:pter-144281984 | ******** | **11, 14, 20**, 22 | 8, 13 | ******** |
| GOS26 |  | ******** | Chr1:120310047-203322572 | 14,20 | 7, 8 13,18 | ******** |
| GOS32 |  | Chr1:pter-143916898  Chr11:62750278-qter  Chr16:31488031-qter | ******** | ******** | ******** | Chr11:pter-21133448 |
| GOS133 |  | Chr4:55411793-qter  **Chr11:59949491-qter**  **Chr14:22979420-qter**  **Chr17:pter-21944094** | ******** | ******** | ******** | Chr11:pter-28094790 |
| GOS235 |  | ******** | ******** | ******** | ******** | ******** |
| GOS439 | FH | ******** | ******** | ******** | 3 | Chr4:92539047- 151164398  Chr9: 84192301-93960316  Chr10:131338133-qter  Chr11:pter-46997739  Chr15:40049073-55776267  Chr16:20085436-53627194  Chr17:66017832-72094821 |
| GOS543 |  | **Chr11:20755575-38582912** | ******** | ******** | ******** | ******** |
| GOS576 |  | **Chr11:75893184-qter**  **Chr16:45086927-qter** | Chr1:143887291-qter  Chr20-43138377-qter | ******** | ******** | Chr6:24916262-32924678 |
| GSO578 |  | Chr11:18845442-41101129  Chr11:88846612-94585583 | ******** | ******** | ******** | ******** |
| GOS589 |  | ******** | Chr1:143887291-qter | ******** | 12 | ******** |
| GOS900 |  | Chr7:pter-55512218  **Chr16:45086927-qter** | Chr1:120307631-qter | ******** | 8, 13, 18, 20 | ******** |
|  |  |  |  |  |  |  |
|  |  |  |  |  |  |  |
|  |  |  |  |  |  |  |
|  |  |  |  |  |  |  |
|  |  |  |  |  |  |  |
|  |  |  |  |  |  |  |
